# Genetic Research on Nicotine Dependence Will Facilitate Public Health

**DOI:** 10.1371/journal.pmed.0020396

**Published:** 2005-11-29

**Authors:** Joe Cubells

**Affiliations:** **1**Emory University School of MedicineAtlanta, GeorgiaUnited States of America

W. D. Hall's cogent Research in Translation article [1] on the folly of attempting to use predictive genetic testing in public-health measures to prevent nicotine dependence is a valuable contribution. Indeed, his arguments against predictive testing can easily be applied to virtually any complex genetic disorder. It is certainly important that we in the medical research community continue to offer such articulate education to clinicians, the press, and society in general.

There is a danger, however, that his arguments will be seized by those who oppose supporting research on the genetics of nicotine dependence and other addictions, to the detriment of public health. For example, Merikangas and Risch [2] have already proposed that addictions and several other complex diseases should be deprived of federal research support in favor of other complex disorders, arguing, “The expensive and laborious tools of molecular genetics [should] be prioritized to those diseases ... that cannot now be treated or prevented with environmental changes [such as] type 1 diabetes, multiple sclerosis, autism and schizophrenia. In contrast, gene hunting for disorders that appear to be highly amenable to environmental modification, such as type 2 diabetes, AIDS, alcohol dependence and nicotine dependence, would have lower priority [for federal research support], even though genes may be involved in their etiology.” Those arguments were picked up by right-wing commentators and trumpeted in high-profile lay outlets such as the New York Times. For example, Humphreys and Satel [3] (the latter a resident scholar at the American Enterprise Institute) cite Merikangas and Risch when they conclude, “Some gene research just isn't worth the money ... [because] major cuts in drug- and alcohol-related harm depend not on genes but on choices by policy makers and individual citizens.” Thus, the myth that addictive behavior is simply a matter of “choice” is made to appear as if it has solid science behind it, when in my view, the only real rationale for opposing genetic research on disorders related to smoking, drinking, overeating, homosexual sex, and other “sinful” behavior derives from the same strain of religious moralism underlying creationism and intelligent design.

Such arguments miss the most important rationale for genetic research on addictions and other environmentally influenced complex disorders. These conditions deserve continued vigorous support from the National Institutes of Health and other sources because genetic research is a powerful tool for pointing us toward new treatments based on improved understanding of the biology of the disorders. Nicotine dependence is an important case in point because current treatments, which consist of psychosocial interventions, and medication therapies such as nicotine replacement and buproprion are, in a word, ineffective: relapse rates following smoking cessation with those strategies, while slightly better than no intervention, usually exceed 80% at one-year follow-up [4].

Genetic research, by providing suggestive evidence for associations to the genes encoding the gamma-amino butyric acid (GABA) B receptor subunit 2 [5], or the cannabinoid-1 receptor [6,7], has already helped light the way toward potentially more effective interventions for millions who struggle to quit smoking, but repeatedly fail. While predictive testing of risk for nicotine dependence based on those genetic findings is quite useless, it remains to be ascertained whether pharmacogenetic profiling will be useful for identifying those most likely to benefit from specific medications (or for that matter, psychosocial interventions), or who would be at risk for harmful side-effects from an otherwise effective drug. While the potential for such profiling has been hyped up in the popular press just as predictive testing has been, we have only to recall the lives saved by understanding the genetic basis of variation in thiopurine methyltransferase activity, in the context of thiopurine chemotherapy for acute lymphoblastic leukemia [8], to convince ourselves of the importance of studying the genetic basis of all common complex diseases, whether partially amenable to environmental prevention or not.
